# Different patterns of depressive symptoms during pregnancy

**DOI:** 10.1007/s00737-017-0738-5

**Published:** 2017-06-08

**Authors:** Sophie E. M. Truijens, Viola Spek, Maarten J. M. van Son, S. Guid Oei, Victor J. M. Pop

**Affiliations:** 1 Department of Obstetrics and Gynaecology, Máxima Medical Centre Veldhoven, and Eindhoven University of Technology, Veldhoven/Eindhoven, The Netherlands; 2 Department of Clinical and Health Psychology, Utrecht University, Utrecht, The Netherlands; 3 Department of Medical and Clinical Psychology, Tilburg University, Tilburg, The Netherlands

**Keywords:** Depression, Depressive symptoms, Pregnancy, EDS, Questionnaire assessments

## Abstract

Recently, the US Preventive Services Task Force has advocated to screen pregnant and postpartum women for depression. However, we questioned the meaning of a single elevated depression score: does it represent just one episode of depression or do these symptoms persist throughout the entire pregnancy? This study assessed depressive symptoms at each trimester in a cohort of 1813 pregnant women and evaluated whether women with different patterns of depressive symptoms showed other characteristics. Depending on the trimester, elevated depression scores were prevalent in 10–15% of the pregnant women. Up to 4% reported persistent symptoms of depression throughout pregnancy. Different patterns of depressive symptoms were observed, for which persistent symptoms were related to other characteristics than incidentally elevated symptoms. Besides a previous history of mental health problems as best overall predictor, incidentally elevated depression scores were related to major life events. Furthermore, persistently depressive symptoms were related to unplanned pregnancy and multiparity. An EDS assessment at 12 weeks of gestation including three additional items (history of mental health problems, unplanned pregnancy and multiparity) enabled us to identify 83% of the women with persistent depressive symptoms. A depression screening strategy in pregnant women should take into account the potential chronicity of depressive symptoms by repeated assessments in order to offer an intervention to the most vulnerable women.

## Background

Depression during pregnancy is assumed to have a wide range of consequences for a woman, her partner and their developing child. The World Health Organization (WHO) has highlighted the importance of perinatal mental health under Millennium Developmental Goal 5, as well as in the global mental health action plan (WHO 2013, 2016). Recently, the lifetime costs of a perinatal depression (both antenatal and postnatal) have been estimated to be over £75,700 and accommodates for the impact on both the mother and her offspring (Bauer et al. 2016). These costs have contributed to the discussion regarding a possible benefit of screening and treatment of depression in the perinatal period. Furthermore, the US Preventive Services Task Force has recently advocated for this screening (O’Connor et al. 2016; Siu et al. 2016). Nonetheless, when positive cases are identified during screening, there should be a clear and precise consensus of how to act accordingly. Should every woman identified with a single assessment be treated? Moreover, can all positive cases be diagnosed with comparable forms of depression and can all cases be treated similarly? It is hypothesised that a single assessment of depression may detect women with scores that have been incidentally elevated due to, for example, the experience of possible major life events during that period of time (Meijer et al. 2014a; Truijens et al. 2015). In this case, it might be questioned whether these women are at risk for postpartum depression and will benefit from intervention. Under other circumstances however, a high score might pertain to a woman who suffers from recurrent or persistent depression. This has raised the question whether women with incidental elevated depressive symptoms differ from those with recurrent depressive symptoms.

The current paper reports on the assessment of depressive symptoms at different trimesters during pregnancy, along with the assessment of potential determinants of depression. The primary aim was to explore the existence of possible patterns of depression throughout gestation, i.e. incidental versus persistent symptoms. The secondary aim was to evaluate whether different patterns of depressive symptoms show different characteristics (demographic, obstetric and psychological).

## Materials and methods

### Participants

From January 2013 to September 2014, pregnant women were invited to participate in the HAPPY study (Holistic Approach to Pregnancy and the first Postpartum Year) (Truijens et al. 2014). The midwives of 17 participating community midwife offices in the South-East region of the Netherlands approached pregnant women during their first antenatal appointment of their first trimester. During the recruitment period of 19 months, the midwives informed 3160 Dutch-speaking Caucasian pregnant women receiving care at their offices. The approached women met the inclusion criteria for participation (e.g. singleton pregnancy, no diagnosis of severe psychiatric illness or endocrine disorder; Truijens et al. 2014). Psychiatric patients were not included as the recruitment took place in primary care, while pregnant women with psychiatric disorders receive care in secondary care (at a ‘combined’ outpatient clinic including a psychiatrist, an obstetrician and paediatrician). At the first antenatal consultation, the midwives made this selection based on the information they gather concerning medical, obstetric and psychosocial history. The design of this study has been described in detail elsewhere (Truijens et al. 2014). Written informed consent to participate was signed by 2275 (72%) of the eligible women.

In the Netherlands, 85% of all pregnant women start antenatal care with the community midwife (The Netherlands Perinatal Registry 2014). The remaining 15% of the pregnant population receives prenatal hospital care from obstetricians from the start of pregnancy, due to the existence of chronic medical conditions such as mental health disorders. Women within the HAPPY cohort are classified as non-psychiatric and healthy pregnant women.

The study was approved by the Medical Ethical Committee of the Máxima Medical Centre Veldhoven and the Psychology Ethics Committee of Tilburg University (protocol number EC-2012.25).

### Procedure

Participants received a set of questionnaires at 12, 22 and 32 weeks of gestation. Of the 2275 women included in the HAPPY study, 41 women did not complete all questionnaires because of foetal loss or preterm delivery, and 60 women (2.6%) had never started the questionnaire assessments for unknown reasons (possibly due to foetal loss). Of the 2174 remaining women who started the HAPPY study, 1813 women (83.4%) returned fully completed questionnaires during all three assessment times.

### Data collection

Participants’ demographic, obstetric, lifestyle and psychological features were obtained at baseline (first trimester questionnaire). We defined a history of mental health problems as reporting the past occurrence of psychological problems such as depression, anxiety, occupational burn-out or job burn-out, e.g. and/or having previously received treatment for mental health problems. The occurrence of major life events was asked at each trimester (first trimester assessment: Did you experience a negative event with a major impact since the start of pregnancy? Second and third trimester assessment: Did you experience a negative event with a major impact since the last questionnaire?) and defined as present when a woman reported at least one life event during any trimester.

The Dutch version of the Edinburgh Depression Scale (EDS) (Cox et al. 1987; Pop et al. 1992) was used to measure symptoms of depression at all trimesters. The EDS consists of ten items with a total score ranging from 0 to 30. Higher scores indicate more symptom severity of depression.

The EDS has been validated in the Netherlands for use during pregnancy with trimester specific cut-off values of ≥11 in the first trimester and ≥10 in the second and third trimester giving the most adequate combination of sensitivity, specificity and positive predictive value for depression during pregnancy (Bergink et al. 2011). The Cronbach’s alphas of the EDS in the current study at the first, second and third trimester were 0.82, 0.83 and 0.83, respectively. A trimester-specific elevated score of the EDS was defined as ‘depression’.

### Statistical methods

Statistical analyses were performed using SPSS (version 22, IBM, Chicago, Illinois, USA). Descriptive statistics were performed to evaluate the prevalence of depression at each trimester. On the basis of the assessments, four different groups were defined as follows. The control group included women with no depression at any trimester and was used as the reference group (group 0). Group 1 encompassed those with an elevated score only once during gestation, and group 2 included those with an elevated score at two time points during pregnancy. Women with an elevated score at all trimesters were defined as persistently (chronically) depressed (group 3). Differences with regard to several characteristics between groups were compared with *χ* ^2^ analyses for categorical data and *t* tests or ANOVA for continuous data (*p* < 0.05, with Bonferroni correction for multiple testing). Finally, three multiple logistic regression analyses (OR, 95% CI) were performed in the control group and a specific subgroup of depressed women with one or more elevated depression scores (yes/no) as the dependent variable. The possible relationship between depression and a set of variables known to be related to depression (demographic features, obstetric features, psychological features) was investigated. Because large sample sizes more easily result in statistically significant differences between groups, effect sizes of associations were calculated (for *t* tests the Cohen’s *d*, ANOVA the η^2^ coefficient).

## Results

The participants’ demographic, obstetric, lifestyle, and psychological features are shown in Table 1.

**Table 1:** Characteristics of the 1813 pregnant women

	Mean (SD) [range]	*n* (%)
Age, in years	30.4 (3.7) [19–43]	
Living together with partner		1786 (98.5)
Paid job		1692 (93.3)
High educational level^a^		1179 (65.0)
Multiparity		903 (49.8)
Unplanned current pregnancy		101 (5.6)
Previous abortion or miscarriage		491 (27.1)
Pre-pregnancy BMI	23.8 (4.0) [16–41]	
Smoking during pregnancy		87 (4.8)
Alcohol consumption during pregnancy		50 (2.8)
Use of antidepressants		18 (1.0)
History of mental health problems		633 (34.9)
Occurrence of MLE during gestation^b^		493 (27.2)

^a^High level of education means at least 16 years of education (college education/university)

^b^ *MLE* major life event(s), reported at either first, second or third trimester assessment

Major life events reported at the first assessment were 1% pregnancy-related events and 4% non-pregnancy related events (of which the most common were loss of family member or friend 1.4%, health-related problems of family/friends 0.8% and job-related events 0.7%). At the second trimester assessment, 2% pregnancy-related and 13% non-pregnancy-related problems were reported (of which the most common were loss of family member or friend 4.4%, health-related problems of family/friends 4.1% and job-related events 2.1%). At the third assessment, 2.8% pregnancy-related life events were reported and 11.4% non-pregnancy-related events (of which the most common were loss of family member or friend 3.8%, health-related problems of family/friends 3.1%, job-related events 1.8% and 1% move or home-related problems). As there is overlap between the categories of life events, and some will have impact for a longer period, we defined major life events as present when a woman reported at least one life event during any trimester, which is the case in 27% of the pregnant women.

### Prevalence of depressive symptoms in the whole sample

**Fig. 1:**
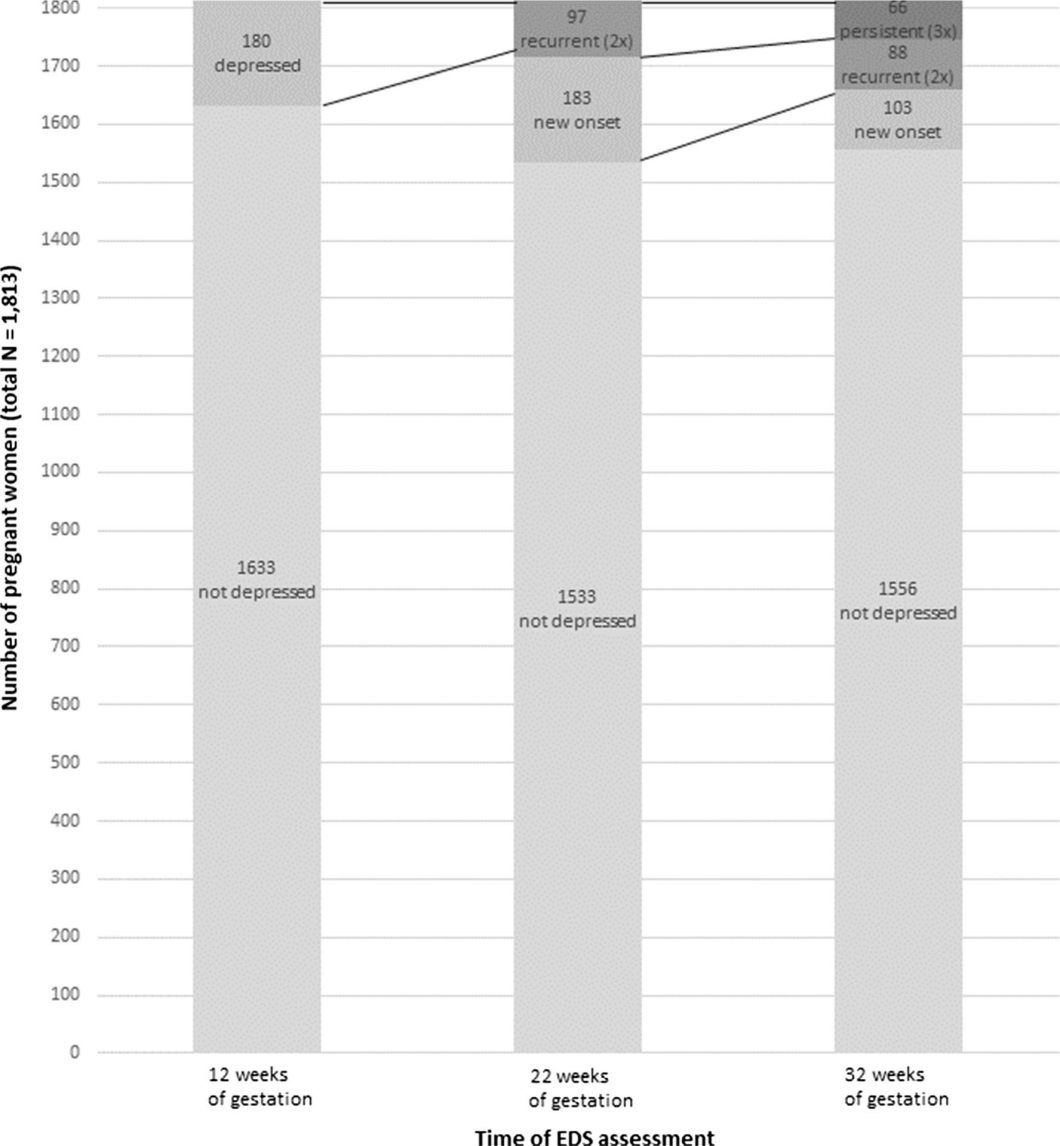
Number of pregnant at each trimester with a score below (not depressed) or above the EDS cut-off (depressed). During the second and third trimester assessments, this was divided by ‘new onset’, ‘recurrent’ or ‘persistent’ depression

Using the trimester specific cut-off values of depression, 466 (25.7%) women of the total group of 1813 women reported an elevated depression score during one or more trimesters, while 1347 (74.3%) did not have a depression at any trimester (control group). The prevalence of depression was 180 (9.9%) in the first trimester, 280 (15.4%) in the second trimester and 257 (14.2%) in the third trimester of pregnancy. Throughout gestation, 66 women (3.6%) reported depression at all trimesters, 281 (15.5%) reported an EDS depression score once, and 119 women (6.6%) reported elevated symptoms of depression at two trimesters (Table 2). Figure 1 shows the number of women with and without an elevated depression score at the first assessment, followed by the number of women with a recurrent (≥2×), persistent (3×) or a new onset depression. The mean (SD) scores of the EDS scores of all these subgroups are shown in Table 2. There were 141 (7.8%) women who reported an EDS score higher than 14 at one or more trimesters.

**Table 2:** Mean EDS scores among different subgroups of the 1813 pregnant women with and without EDS scores above the trimester specific cut-offs

		Subgroups of women with number of EDS scores above the cut-off (↑)				
	Total	0 = Controls^a^	1 = Once ↑	2 = Twice ↑	3 = Persistent ↑	
	*N* = 1813	*n* = 1347	*n* = 281	*n* = 119	*n* = 66	ANOVA
Time of assessment	Mean (SD)	Mean (SD)	Mean (SD)	Mean (SD)	Mean (SD)	F (df = 3)
First trimester	4.39 (4.19)	2.91 (2.59)	6.90 (4.23)	9.66 (4.08)	14.50 (3.51)	529.0*
Second trimester	5.13 (4.23)	3.48 (2.61)	8.35 (3.92)	11.57 (3.26)	14.59 (3.11)	684.7*
Third trimester	5.01 (4.22)	3.39 (2.63)	8.10 (3.83)	11.04 (3.66)	14.67 (3.47)	637.1*

*p < .001 (after Bonferroni correction), η^2^ all >0.46

^a^group 0 = control group with no elevated EDS score

As confirmed by Table 2, the mean EDS scores significantly differed between the groups. In group 0, 1 and 2, the mean depression score significantly increased from 12 to 22 weeks of gestation (paired samples *t* tests, *p* < 0.01) and remained stable between 22 and 32 weeks. Women with EDS scores persistently above the cut-off (group 3) had more stable high mean scores during all assessments.

Analyses of variance (ANOVA) confirmed that women in the control group had significantly lower mean EDS scores compared to the other three groups at all trimesters: F (3; 1, 809) = 529.0 at 12 weeks, F (3; 1, 809) = 684.7 at 22 weeks, F (3; 1, 809) = 637.1 at 32 weeks, respectively (*p* < .001 after Bonferroni correction, η^2^ all >0.46 which indicates a large effect size according to Cohen (1988)). Women who had a score above the cut-off only once (group 1) also had mean scores at all trimesters which were significantly higher compared to controls (group 0): *t*(1626) = 15.2 at 12 weeks, *t*(1626) = 19.9 at 22 weeks, *t*(1626) = 19.6 at 32 weeks, respectively (*p* < 0.001 after Bonferroni correction, Cohen’s *d* effect size of 1.13, 1.46 and 1.43, respectively, which all indicate a very large effect size (d > .80) (Cohen 1992).

Finally, Table 3 shows the results of three separate multiple logistic regression analyses with elevated depression scores during pregnancy (yes/no) as the dependent variable, each time combining the control group (group 0) with groups 1, 2, and 3 separately: groups 0 + 1 (controls with an elevated EDS score once, *n* = 1628), groups 0 + 2 (controls with an elevated EDS score twice, *n* = 1466) and groups 0 + 3 (controls with persistently elevated EDS score, *n* = 1413). In all three regression analyses, the independent effects of a similar set of possible determinants were investigated. These determinants included age, parity, educational level, having no paid job, unplanned pregnancy, the occurrence of major life events during pregnancy and a history of mental health problems earlier in life.

**Table 3:** Three multiple logistic regression analyses with EDS score above the cut-off (yes/no) as dependent variable in three different groups of pregnant women taking into account the number of elevated depression scores during gestation (controls = no elevated depression score)

	Controls (*n* = 1347) vs once EDS↑(*n* = 281)		Controls (*n* = 1347) vs twice EDS↑ (*n* = 119)		Controls (*n* = 1347) vs three times EDS↑ (*n* = 66)	
	OR	95% CI	OR	95% CI	OR	95% CI
Higher age	0.98	0.94–1.02	0.92	0.87–0.98**	0.93	0.86–1.00
Multiparity	1.21	0.91–1.60	1.49	0.98–2.26	2.30	1.28–4.12**
Lower education	1.07	0.80–1.43	1.15	0.75–1.76	1.41	0.80–2.49
No paid job	1.51	0.92–2.46	1.26	0.62–2.55	1.75	0.79–3.91
Unplanned pregnancy	1.62	0.92–2.85	2.39	1.17–4.89*	5.13	2.43–10.85**
Occurrence of MLE during pregnancy	1.66	1.25–2.20**	1.65	1.09–2.49*	1.63	0.93–2.83
History of mental health problems	2.41	1.83–3.16**	3.18	2.13–4.74**	2.92	1.71–4.99**

*EDS*↑ Edinburgh Depression Scale score above the cut-off, *MLE* major life events

*p < .05; **p < .01

As seen in Table 3, there were different characteristics of depressive symptoms in the various subgroups. In the first analysis, the controls and the women with a depression score above the cut-off at one point during pregnancy were analysed. Results showed that major life events during pregnancy (OR 1.66, 95% CI 1.25–2.20) and a history of mental health problems (OR 2.41, 95% CI 1.83–3.16) were two determinants significantly related to depressive symptoms. In the second analysis, with controls versus two assessments of elevated depression scores, two additional variables were related to depression: age (OR 0.92, 95% CI 0.87–0.98) and unplanned pregnancy (OR 2.39, 95% CI 1.17–4.89). In the third analysis, comparing no elevated depression score with persistently elevated depression score, the occurrence of MLE was not significantly related to depression. Apart from unplanned pregnancy (OR 5.13, 95% CI 2.43–10.85) and a history of mental health problems (OR 2.92, 95% CI 1.71–4.99), multiparity (OR 2.30, 95% CI 1.28–4.12) was significantly and independently related to (persistent) depression.

A substantial higher proportion of the women with a persistently elevated depression score (group 3) reported a history of mental health problems, compared to the remainder of the total sample (57.6 vs. 34.1%, *χ* ^2^(1) 15.5, *p* < 0.001).

When focusing on the 180 (9.9%) women with an EDS score above the cut-off at the first assessment, 97 of these women reported a high depression score again at the second trimester, and 66 reported their third (=persistent) high depression score at the third trimester. As our goal is to identify most of these vulnerable women early in pregnancy, we tested whether a selection could be made based on the presence of at least one of the determinants of a persistent depression (unplanned pregnancy, history of mental health problems and multiparity), of which the information is already known at the beginning of pregnancy. Of the 180 women with a high depression score at the first trimester, 150 women reported at least one of the determinants of persistent depression (unplanned pregnancy, history of mental health problems and multiparity). Statistical analyses showed that 55 of these 150 women indeed reported persistent depression scores. The total number of women with a persistent depression was 66, so 55 of the 66 (83.3%) women with persistent depression scores could already be detected in the first trimester. The other 11 of the 66 could not be identified with just the information of the EDS score and the three baseline questions at the first trimester. In terms of positive and negative predictive values, the positive predictive value (true positive/(true positives + false positives) = 55/150) is 36.7%. However, as it is more important that women with a persistent depression should not be missed, the negative predictive value (NPV) is even more important. The negative predictive value (true negatives/(true negatives + false negatives) = 19/30) is 63.3%.

## Discussion

The current study shows that 26% of women belonging to a healthy pregnant population reported depressive symptoms at least once during gestation. The trimester-specific prevalence of depressive symptoms varies from 10 to 15%. Up to 4% of the women report persistent depressive symptoms at all trimesters and 58% of these women report a previous history of mental health problems. Compared to controls, pregnant women with a single reported high EDS score also show significantly higher mean EDS scores at the other trimesters. The frequency of high scores showed that different characteristics were associated with depression. In particular, an unplanned pregnancy was related to persistent depression. At 12 weeks of gestation, the group of women with persistent depressive symptoms can be identified in up to 83% of the cases using a simple set of four items: EDS score above the cut-off, previous history of mental health problems, unplanned pregnancy and multiparity.

The obstetric characteristics (e.g. parity, previous miscarriage, mean age pregnant women) of this sample are representative for the Dutch pregnant population (The Netherlands Perinatal Registry 2014). The results can suggest that there is an underestimation of the prevalence and severity of depressive symptoms. This is deduced from the finding that one in four women present elevated symptoms of depression. Of importance is the fact that, as part of the HAPPY exclusion criteria, women with any known psychiatric problems were not included in this study (5–10% of the pregnant population among whom mood disorders are most common (Andersson et al. 2003; O’Keane et al. 2006) and the use of SSRI among 2–5% of the pregnant women (e.g. Ververs et al. 2006; Bakker et al. 2008; Quispel et al. 2012). This suggests that in the total pregnant population, this value might be even higher.

Our reported prevalence rate of depressive symptoms ranging from 10 to 15% throughout gestation is comparable to recent literature, (Allbaugh et al. 2015; Ashley et al. 2016; Castro e Couto et al. 2016; Lara et al. 2015; Quispel et al. 2014) as well as our mean EDS scores (Bergink et al. 2011; Meijer et al. 2014b; Rallis et al. 2014). These studies, however, did not focus on the characteristics of women with persistent symptoms of depression. We defined persistent depression as having three consecutive elevated scores of the EDS. We were aware that these EDS scores refer to symptoms occurring during a period of the last 7 days. Theoretically, it is possible that between these assessments, the women may have had lower scores (below cut-off). The current study demonstrated, however, that a single high EDS score during pregnancy was already associated with significantly higher mean scores throughout gestation in comparison to those who did not have an elevated EDS score. In women with persistent depression, different characteristics are of importance compared to women with incidentally elevated EDS scores. Apart from a previous history of mental health problems that have repeatedly been reported in the literature (Dudas et al. 2012; Lancaster et al. 2010; Martini et al. 2015), persistently high depressive symptoms were related to an unplanned pregnancy and multiparity. The association between unplanned pregnancy and perinatal depression was recently outlined by a systematic review of Abajodir et al. (2016). It was concluded that the prevalence of depression during pregnancy is twofold in women with unintended pregnancy, especially in developed countries. The current study shows an OR of 5.13 for unplanned pregnancy to report persistent depression. The direction of this association is still unclear, since depressed women may also have high rates of unintended pregnancies (Abajodir et al. 2016).

In 1988, Lewinsohn and colleagues already described a relationship between depression and living with young children, but there are inconclusive results about the relationship between depression and parity (Lancaster et al. 2010; Lewinsohn et al. 1988). Since our study found this association only in the group with persistently depressive symptoms, it might be speculated that it is pattern specific: the presence of other (most of the time very young) children is a stressor only in a highly vulnerable group. It should be noted that in the current study, women with persistent depression reported a previous history of mental health problems twice as often.

In Western countries, the discussion about the relevance of screening for depression in the general population and in perinatal women in particular has risen recently (US Preventive Services Task Force program, O’Connor et al. 2016). With regard to pregnancy screening, the current study showed that up to 60% of the 466 cases reported only a single episode of depressive symptoms, for which a major life event was a predominant determinant. Further evaluation will show whether focusing on the remaining 40% of the women with a high EDS score at two or more periods of time during pregnancy will target the most vulnerable group, to which interventions should preferentially be offered. Moreover, adding three questions to the EDS screening instrument at 12 weeks of gestation (previous history of mental health problems, unplanned pregnancy and multiparity) will enable health professionals to identify up to 83% of the highly vulnerable women. This might contribute to a better cost-benefit ratio when intervention is implemented.

The key strength of this study is the longitudinal design that includes data of depressive symptoms at all trimesters of pregnancy from a large sample of women. This large sample enabled us to discriminate different and sufficiently large subgroups of women with various depression patterns which in turn made it possible to discriminate a variety of characteristics related to different patterns of depressive symptoms. The EDS questionnaire, with trimester-specific cut-off values, was used and validated against a psychiatric diagnostic interview conducted with Dutch pregnant women living in the same Dutch region and who had similar characteristics (Bergink et al. 2011).

To check whether partial non-response is related to depression, the available data between the complete cases and the (partial) non-responders were compared. The proportion of women with a depression according to the EDS did not differ between the completers and the non-responders at 12 and 32 weeks of gestation. Although at 22 weeks of gestation the differences between completers and non-responders was statistically significant (15.4 versus 23.4%, χ^2^ = 7.0 *p* < 0.01), the effect size was rather low (Cramer’s V = 0.06) and suggests little to no clinical relevance.

A limitation is the large number of highly educated women participating in this study compared to the representation of the general Dutch female population between 25 and 35 years of age in 2014 (65 versus 52%) (Statistics Netherlands 2016). The trend of over-representing highly educated participants has generally been evident in scientific research but can also be explained by the fact that only Dutch-speaking women of European descent were included. These limitations may limit the generalizability of the findings of the present study.

What is the clinical relevance of the current study? One single elevated score overestimates the number of vulnerable women since one incidentally elevated EDS score can be a temporary effect of, for instance, the occurrence of major life events during pregnancy. A recommendation for clinical practice would be that a depression screening strategy in pregnant women should take into account possible chronicity of depressive symptoms by repeated assessment in order to offer interventions to the most vulnerable women. An elevated EDS score should be placed in the context of repeated measurements and other determinants to focus on women with more ‘chronic’ depressive symptoms. We recommend to start screening in the first trimester, so that foetal development can benefit most from early intervention when mental health problems are detected. In the case of an elevated EDS score, we recommend the completion of the following steps: gather more contextual information (including information about history of mental health, unplanned pregnancy and parity), implement a watchful waiting period to avoid stigmatization and medicalization, take some time for natural recovery and repeat an EDS assessment during the next consultation (2 to 4 weeks later). The frequent health check-ups during pregnancy are suitable for repeated assessments of depression. Furthermore, it is important to discuss this topic with each woman (and her partner if she agrees) in the context of shared-decision making. This is particularly of importance when a possible referral or intervention will take place. As was recently stated in a *Lancet* editorial, perinatal mental health screening needs to be completed through sensitive enquiry in the context of a broader conversation about the physical and mental health well-being of mothers (Lancet editorial 2016). A selection based on a vulnerability profile, repeated EDS assessments and patients’ conviction might contribute to a better cost-benefit ratio when interventions are offered. With a preference for non-medical interventions, there are several plausible psychological interventions that can be applied, such as counselling, cognitive behavioural therapy (CBT) and interpersonal therapy. Another intervention of growing interest is mindfulness-based training, a much less stigmatizing intervention compared to psychological treatment. A publication in *The Lancet* showed mindfulness-based intervention to be equally effective as antidepressant treatment in patients with recurrent depression (Kuyken et al. 2015). Moreover, several types of mental health interventions are web-based nowadays (including online mindfulness training (Krusche et al. 2012; Cavanagh et al. 2013; Spijkerman et al. 2016) and making them highly accessible to pregnant women.

In conclusion, this study shows that there are different patterns of depressive symptoms. Persistent depressive symptoms are related to other characteristics than incidentally elevated symptoms. Focusing on the group at risk could economize intervention resources while avoiding medicalization of women with only a single elevated depression score.
